# Supplementary material for: Biochemical markers after the Norseman Extreme Triathlon
Source: PLoS One. 2020 Sep 23;15(9):e0239158. doi: 10.1371/journal.pone.0239158 (PMC7510962; doi:10.1371/journal.pone.0239158)
Supplement: S1 Table — (DOCX) [file pone.0239158.s001.docx]

**S1 Table. Included samples.**

|  | Ironman 2016 | Ironman 2018 | Ironman 2019 | Olympic 2018 | Total |
| --- | --- | --- | --- | --- | --- |
| ALT | - | 27 | 32 | 15 | 74 |
| AST | - | 27 | 31 | 15 | 73 |
| Ca^2+^ | - | 27 | 32 | 14 | 73 |
| CK | 38 | 27 | 32 | 15 | 112 |
| Creatinine | 38 | 27 | 31 | 15 | 111 |
| CRP | 38 | 27 | 31 | 15 | 111 |
| Hb | - | 28 | 9 | 15 | 52 |
| K^+^ | 38 | 27 | 25 | 15 | 105 |
| Mg^2+^ | - | 27 | 26 | 15 | 68 |
| Na+ | 38 | 27 | 32 | 15 | 112 |
| NT-proBNP | 38 | 27 | 29 | 13 | 107 |
| T3 | - | 27 | 31 | 15 | 73 |
| T4 | - | 27 | 31 | 15 | 73 |
| Thrombocytes | - | 28 | 9 | 15 | 52 |
| TSH | - | 27 | 31 | 15 | 73 |
| WBC | - | 28 | 9 | 15 | 52 |

Table of included analysis of complete sets of samples (pre, after and post-day) per year and race.

Hb, Hemoglobin; WBC, White Blood Cells; CRP, C-reactive protein; AST, Aspartate Aminotransferase; ALT, Alanine Aminotransferase; CK, Creatinine Kinase; NT-proBNP, N-terminal pro Brain Natriuretic Peptide; TSH, Thyroid Stimulating Hormone; T3, Triiodothyronine; T4, Thyroxine.
