# Supplementary material for: Biochemical markers after the Norseman Extreme Triathlon
Source: PLoS One. 2020 Sep 23;15(9):e0239158. doi: 10.1371/journal.pone.0239158 (PMC7510962; doi:10.1371/journal.pone.0239158)
Supplement: S3 Table — (DOCX) [file pone.0239158.s003.docx]

**S3 Table. Results for only females.**

| Variable | Race | Baseline | Finish | Day after |
| --- | --- | --- | --- | --- |
| Hb | N | 14 [13.6 - 14.3] | 13.4 [12.9 - 13.7] | 13 [12.7 - 13.3] * |
| (g/100 mL) | O | 13.6 [13.2 - 13.9] | 13.1 [12.9 - 13.8] | 12.7 [12.5 - 13.4] |
| WBC | N | 4.1 [3.7 - 4.7] | 12.6 [10.7 - 14.2] *** | 6.2 [5.8 - 9.4] ** |
| (10^09 /L) | O | 3.2 [2.7 - 4.6] | 10.3 [6.5 - 10.6] | 3.4 [3.4 - 5.7] |
| Thrombocytes | N | 233 [206 - 242] | 256 [236 - 267] | 238 [230 - 250] |
| (10^09 /L) | O | 170 [163 - 216] | 191 [172 - 232] | 157 [154 - 194] |
| CRP | N | 1 [0 - 1] | 4 [2 - 25.2] *** | 30 [20 - 70.2] *** |
| (mg/L) | O | 1 [1 - 1] | 1 [1 – 1] | 1 [1 - 2] |
| Na | N | 140 [139 - 141] | 138 [137 - 140] ** | 140 [139 - 141] |
| (mmol/L) | O | 140 [140 - 140] | 139 [139 - 140] | 140 [140 - 142] |
| K | N | 4.4 [4.1 - 4.6] | 4.2 [4 - 4.4] * | 4.0 [3.8 - 4.2] *** |
| (mmol/L) | O | 4.1 [4.1 - 4.2] | 4.5 [4.4 - 4.6] | 4.1 [4 - 4.2] |
| Ca | N | 2.36 [2.31 - 2.43] | 2.45 [2.34 - 2.48] | 2.29 [2.27 - 2.36] |
| (mmol/L) | O | 2.41 [2.40 - 2.42] | 2.45 [2.41 - 2.45] | 2.37 [2.37 - 2.41] |
| Mg | N | 0.80 [0.76 - 0.85] | 0.89 [0.85 - 0.93] * | 0.85 [0.80 - 0.88] |
| (mmol/L) | O | 0.79 [0.79 - 0.80] | 0.67 [0.63 - 0.68] | 0.79 [0.78 - 0.81] |
| Creatinine | N | 68 [60 - 71] | 80 [75 - 89] *** | 75 [70 - 80] ** |
| (umol/L) | O | 67 [65 - 69] | 75 [75 - 77] | 66 [63 - 69] |
| AST | N | 27 [25 - 28] | 87 [73 - 129] *** | 100 [79 - 156] *** |
| (U/L) | O | 20 [19 - 24] | 23 [23 - 30] | 28 [26 - 32] |
| ALT | N | 29 [26 - 34] | 44 [37 - 48] *** | 49 [39 - 60] *** |
| (U/L) | O | 32 [28 - 36] | 33 [28 - 42] | 31 [27 - 38] |
| CK | N | 127 [108 - 189] | 2050 [1520 - 3510] *** | 2100 [1280 - 3290] *** |
| (U/L) | O | 66 [65 - 94] | 152 [146 - 170] | 197 [190 - 267] |
| NT-proBNP | N | 51 [28.8 - 72] | 820 [585 - 1500] *** | 360 [270 - 590] *** |
| (ng/L) | O | 22 [21 - 29] | 81 [60 - 91] | 103 [63 - 126] |
| TSH | N | 2 [1.7 - 2.7] | 3.1 [2.2 - 4.1] | 2.4 [1.9 - 2.9] |
| (mU/L) | O | 3.2 [2.4 - 3.6] | 2.1 [1.5 – 3.0] | 1.8 [1.3 - 1.9] |
| T3 | N | 4.3 [3.9 - 4.5] | 4.2 [3.7 - 4.5] | 4.25 [3.8 - 4.7] |
| (pmol/L) | O | 4.9 [4.4 - 5.2] | 4.6 [4.1 - 4.6] | 4.2 [3.9 - 4.3] |
| T4 | N | 14.4 [14.2 - 16.2] | 17.2 [15.9 - 19.2] *** | 15.6 [15.0 - 17.5] * |
| (pmol/L) | O | 15.7 [15.7 – 17.0] | 16.3 [15.4 - 17.8] | 15.4 [15.2 - 16.6] |

Results for females only, presented in the same format as all results in table 2. The results are given as median [1. quartile – 3. quartile] for Norseman (N) and Olympic (O) triathlons. Measurements were performed before start as baseline, at finish and the day after races. Statistical difference from baseline where tested with Wilcox Ranked Sum tests.

N, Norseman; O, Olympic triathlon; Hb, Hemoglobin; WBC, White Blood Cells; CRP, C-reactive protein; AST, Aspartate Aminotransferase; ALT, Alanine Aminotransferase; CK, Creatinine Kinase; NT-proBNP, N-terminal pro Brain Natriuretic Peptide; TSH, Thyroid Stimulating Hormone; T3, Triiodothyronine; T4, Thyroxine.

* *p* value < 0.05

** *p* value < 0.01

*** *p* value < 0.001
