# Supplementary material for: Biochemical markers after the Norseman Extreme Triathlon
Source: PLoS One. 2020 Sep 23;15(9):e0239158. doi: 10.1371/journal.pone.0239158 (PMC7510962; doi:10.1371/journal.pone.0239158)
Supplement: S4 Table — (DOCX) [file pone.0239158.s004.docx]

**S4 Table. Differences between genders.**

| **Variable** | **Race** | **Baseline** | **Finish** | **Day after** |
| --- | --- | --- | --- | --- |
| **Hb** | N | < 0,001 | < 0,001 | < 0,001 |
|  | O | < 0,05 | < 0,05 | 0,070 |
| **CRP** | N | 0,51 | 0,23 | 0,29 |
|  | O | - | 0,37 | - |
| **WBC** | N | 0,80 | 0,27 | 0,57 |
|  | O | 0,89 | 0,61 | 0,83 |
| **Thrombocytes** | N | 0,17 | 0,47 | 0,43 |
|  | O | 0,63 | 0,61 | 0,25 |
| **Na** | N | 0,78 | 0,88 | < 0,05 |
|  | O | 0,01 | 0,55 | < 0,05 |
| **K** | N | < 0,05 | < 0,01 | < 0,05 |
|  | O | < 0,05 | < 0,05 | 0,110 |
| **Ca** | N | 0,81 | < 0,05 | 0,14 |
|  | O | 0,31 | 0,94 | 0,16 |
| **Mg** | N | 0,52 | 0,06 | 0,31 |
|  | O | 1,00 | 0,15 | 0,06 |
| **Creatinine** | N | < 0,01 | < 0,001 | < 0,001 |
|  | O | < 0,05 | < 0,05 | < 0,05 |
| **AST** | N | 0,71 | 0,11 | 0,41 |
|  | O | 0,77 | 0,56 | 0,89 |
| **ALT** | N | 0,55 | 0,22 | 0,85 |
|  | O | 0,66 | 0,61 | 0,61 |
| **CK** | N | < 0,05 | < 0,01 | 0,08 |
|  | O | 0,10 | 0,08 | 0,05 |
| **NT-proBNP** | N | < 0,001 | < 0,001 | < 0,001 |
|  | O | 0,93 | 0,57 | 0,80 |
| **TSH** | N | < 0,01 | < 0,001 | < 0,05 |
|  | O | 0,77 | 0,51 | 1,00 |
| **T4** | N | 0,01 | 0,27 | 0,29 |
|  | O | 0,94 | 0,89 | 0,94 |
| **T3** | N | < 0,001 | < 0,05 | < 0,01 |
|  | O | 0,13 | < 0,05 | < 0,05 |

P-values from Wilcoxon Rank Sum tests between the sexes for each biochemical marker for each time.

N, Norseman; O, Olympic triathlon; Hb, Hemoglobin; WBC, White Blood Cells; CRP, C-reactive protein; AST, Aspartate Aminotransferase; ALT, Alanine Aminotransferase; CK, Creatinine Kinase; NT-proBNP, N-terminal pro Brain Natriuretic Peptide; TSH, Thyroid Stimulating Hormone; T3, Triiodothyronine; T4, Thyroxine.
