# Supplementary material for: Biochemical markers after the Norseman Extreme Triathlon
Source: PLoS One. 2020 Sep 23;15(9):e0239158. doi: 10.1371/journal.pone.0239158 (PMC7510962; doi:10.1371/journal.pone.0239158)
Supplement: Attachment — Submitted filename: Response to Reviewers.docx [file pone.0239158.s006.docx]

# Response to Reviewers

Firstly we would like to thank you for considering our article and thank all the reviewers for valuable feedback. We have done our best to improve the article and hope you will find it worthy for publication in PLOS ONE. Response to each reviewer follows. A revised manuscript is uploaded as well as a version with track changes from the previously submitted version.

Best regards,

Christoffer Nyborg

*Corresponding author*

## Response to Journal requirements

1. Updates have been made to ensure that your manuscript meets PLOS ONE's style requirements.
2. Updated Funding Statement and Competing interests are given in the cover letter.

## Response to Reviewer #1

- We have changed to a more ‘neutral’ title.
- The abstract is revised based on your and the other reviewers input.
- Thanks for relevant major literature. This is included in the introduction and discussion.
- Changes meters to m in the characteristics table.
- References are added were requested.
- The term “extreme exercise” is revised to “prolonged exercise” as suggested.
- The need for reference values for medical teams is the main gap in literature and is described.
- The participants received e-mails 1-2 months prior to the events. This is included in the methods chapter.
- Hypotheses and aim has been clarified in the introduction.
- The introduction has been simplified and made more specific.
- Sugested grammatical changes have been altered
- A paragraph with strengths and limitations has been added. Practical applications have been clarified in the introduction and conclusion.

## Response to Reviewer #2

- The concerns about time between measurements and the issue with swimming in cold water are addressed in a new paragraph with strengths and limitations.
- We agree that much is known about the physiological impact of prolonged exercise and have reformulated our introduction. We have also included the major literature you have made us aware of.
- The word “injury” is removed due to revision of the abstract. The word accident is used in the introduction, as this is a major concern in large scale Ironman races as Norseman.
- We did intravenous blood samples form the antecubal vein. This is clarified in the method section.
- We have used the word “changes” as some of the measured electrolytes could potentially also shown decreased values. However all clinically significant changes that we found where increased values.
- We have chosen to keep the absolute values in the results chapter. The reasons are that the aim of our article is to provide reference values for clinicians and therefore the absolute value is our primary goal. As well as all of these biomarkers and their values should be familiar for medical personnel and it is absolute values that are used in the clinic, not relative change. However the relative change can easily bed deduced from the tables and the raw data if warranted.
- The practical usage of the data is addressed.
- SI units have been corrected. We chose to change the scale to cm due to a rounding-off issue addressed in the review process.
- We did not find any major changes between other papers describing biomarkers after Ironman triathlons.
- The statistics section is simplified and revised according to input from you and other reviewers.
- We changed to utilize “Baseline”, “Finish” and “Day After” throughout the article to simplify the reading of the time points.
- It was R-values in the correlations; this is clarified in the statistics section.
- You spotted a honest mistake about the value of the WBC at finish line. By mistake it was switched with the Hb value. This is corrected.
- We decided to keep individual connected values in Figure 2. We also would like to keep separation of the genders by color. We don’t believe gender differences to be a major concern in the article, but there are some differences. This is addressed in the new strengths and limitation section and made available to study through the supplemental information provided. There are also different reference values between the sexes for some of the biomarkers. Therefore we would like to fully visualize the data for readers familiar with the biomarkers and these gender differences (e.g. NT-proBNP) so they can be evaluated. Connected individual values give both the size of measure, spread and the direction of change (the main parameter in most statistical tests). So we believe this figure gives valuable information to certain readers.
- It can often be hard to understand the clustered connection in multiple correlations. We therefore believe the visualized matrix give valuable insight. While the text give exact coefficients and p-values.
- We have uniformed the use of leukocyte/leucocyte.
- The text concerning IL-6 and CRP is clarified. IL-6 is produced by leucocytes at site of pathology. IL-6 then stimulates CRP production in the liver.

## Response to Reviewer #3

- Major literature that you provided is now included.
- Hypothesis and aim is clarified.
- The inclusion criteria are clarified. We had no exclusion criteria.
- References are provided were warranted.
